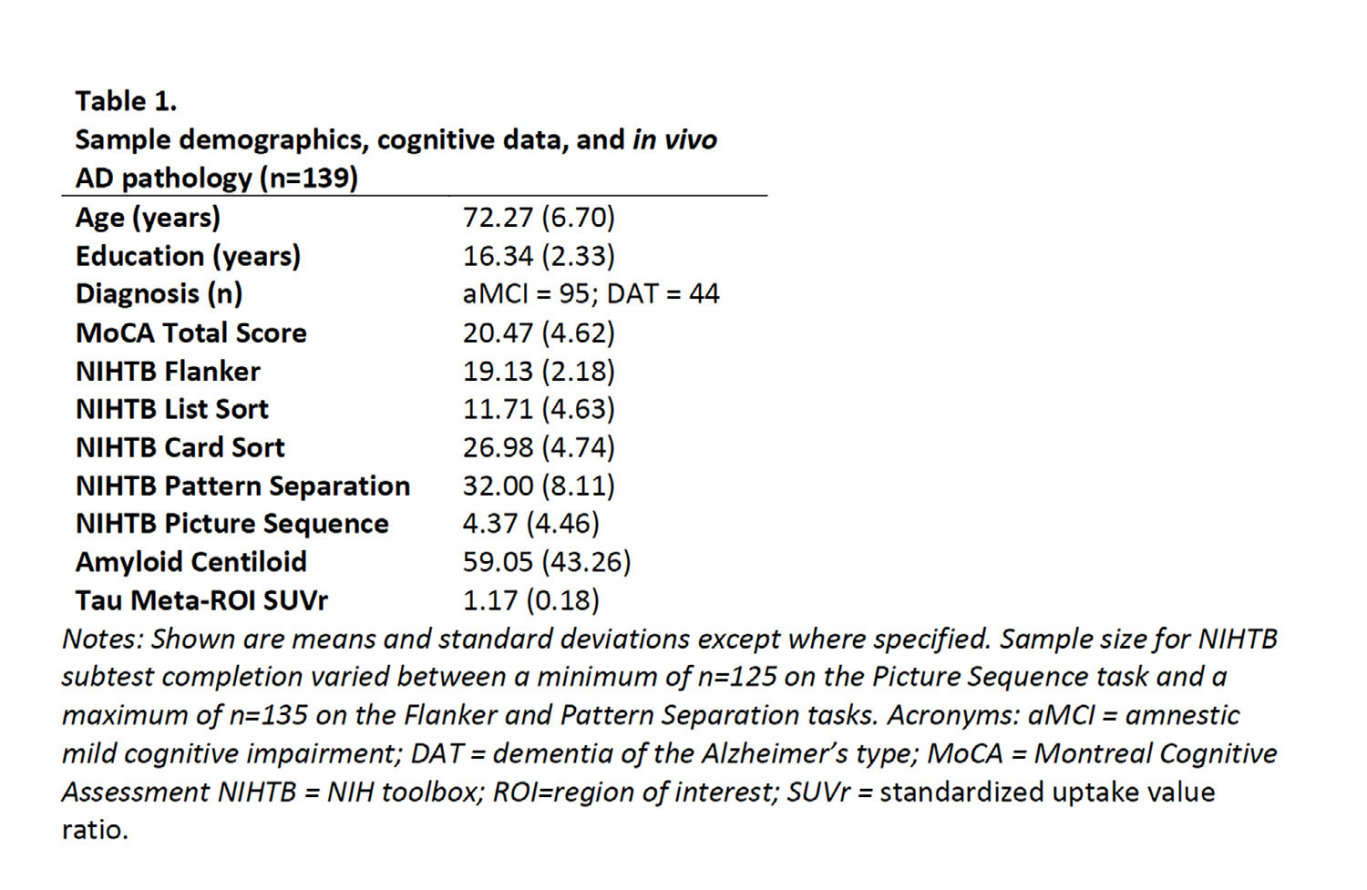# Relationships Between NIH Toolbox Cognitive Battery Subtests and *In Vivo* Amyloid and Tau in People with Amnestic Mild Cognitive Impairment and Dementia of the Alzheimer’s Type

**DOI:** 10.1002/alz.091809

**Published:** 2025-01-03

**Authors:** Joshua T Fox‐Fuller, Kenneth Petscavage, Ginny Rogers, Robert A. Koeppe, Roger L. Albin, Alexandru D Iordan, Benjamin M. Hampstead, Annalise Rahman‐Filipiak

**Affiliations:** ^1^ Research Program on Cognition and Neuromodulation‐Based Interventions, University of Michigan, Ann Arbor, MI USA; ^2^ Michigan Alzheimer’s Disease Research Center, Ann Arbor, MI USA; ^3^ Division of Nuclear Medicine, University of Michigan, Ann Arbor, MI USA; ^4^ University of Michigan, Ann Arbor, MI USA; ^5^ VA Ann Arbor Healthcare System, Ann Arbor, MI USA; ^6^ Michigan Alzheimer’s Disease Research Center ‐ University of Michigan, Ann Arbor, MI USA

## Abstract

**Background:**

The computerized NIH Toolbox Cognition Battery (NIHTB‐CB) was designed to assess cognitive functioning across the lifespan. Previous studies demonstrated that NIHTB‐CB measures discriminate between healthy controls (HCs), individuals with amnestic mild cognitive impairment (aMCI), and individuals with dementia of the Alzheimer’s type (DAT). Scores on NIHTB‐CB tasks also correspond with performance on well‐validated neuropsychological measures of the same cognitive domains. We are among the first to evaluate how NIHTB‐CB subtest performances and *in vivo* Alzheimer’s disease (AD) pathology are related in people with aMCI and DAT.

**Method:**

As part of a larger clinical trial in the Research Program on Cognition and Neuromodulation Based Interventions, 139 participants with aMCI or DAT (Table 1) underwent neuropsychological assessment, including five iPad NIHTB‐CB subtests: Flanker, List Sort, Card Sort, Pattern Separation, and Picture Sequence. Participants also completed positron emission tomography (PET) scans of amyloid and tau. Amyloid PET scans were normalized to the centiloid value, and tau PET standardized uptake value ratios (SUVr) were merged into a bilateral meta‐region‐of‐interest based on Braak staging. Stepwise linear regressions were used to evaluate the relationship between age and education with NIHTB‐CB subtests (Model 1), as well as *in vivo* AD pathophysiology with NIHTB‐CB subtests beyond age and education (Model 2).

**Result:**

*In vivo* amyloid was significantly related to List Sort (β = ‐0.31, *p*<.001, Δ*R^2^ =* .09) and Card Sort performance (β = ‐0.25, *p* = .004, Δ*R^2^ =* .06), explaining significantly more subtest variance beyond age and education. *In vivo* tau was significantly related to performance on all NIHTB‐CB subtests and explained significantly more subtest variance than demographic factors (Flanker: β = ‐0.25, *p* = .004, Δ*R^2^ =* .06; List Sort: β = ‐0.40, *p*<.001, Δ*R^2^ =* .15; Card Sort: β = ‐0.32, *p*<.001, Δ*R^2^ =* .10; Pattern Separation: β = ‐0.18, *p* = .04, Δ*R^2^ =* .03; Picture Sequence: β = ‐0.23, *p* = .01, Δ*R^2^ =* .05).

**Conclusion:**

*In vivo* AD pathophysiology is related to NIHTB‐CB subtest performances in individuals with aMCI or DAT and explains significantly more NIHTB‐CB subtest variance than demographic factors alone. This work adds growing support to the relationship between NIHTB‐CB subtests and *in vivo* AD biomarkers.